# Living in a rural area as a risk factor for worst outcomes in penile cancer

**DOI:** 10.1590/S1677-5538.IBJU.2021.99.15

**Published:** 2021-06-15

**Authors:** Lina Garcia, Leonardo Oliveira Reis, Herney Andrés García-Perdomo

**Affiliations:** 1 Universidad del Valle School of Medicine UROGIV Research Group Cali Colombia UROGIV Research Group, School of Medicine, Universidad del Valle, Cali, Colombia;; 2 Universidade Estadual de Campinas UroScience CampinasSP Brasil UroScience, Universidade Estadual de Campinas – UNICAMP, Campinas, SP, Brasil;; 3 Pontifícia Universidade Católica de Campinas Departamento de Urologia CampinasSP Brasil Departamento de Urologia, Pontifícia Universidade Católica de Campinas, PUC-Campinas, Campinas, SP, Brasil;; 4 Universidad del Valle School of Medicine Department of Surgery Cali Colombia Division of Urology/Urooncology. Department of Surgery. School of Medicine. Universidad del Valle. Cali, Colombia

## COMMENT

Penile cancer, especially squamous cell carcinoma, is considered a rare genitourinary malignancy. In developed countries like the US and some countries in Europe, the incidence could be as low as 0.4 to 0.6% (1), meanwhile, in places like South America, South-East Asia, and Africa, the incidence could be as high as 1 to 2% of male cancers (2). Brazil is known for having the third higher incidence of penile cancer worldwide (3), and Maranhão, a State in the northeast of this country, has the highest worldwide incidence (4). The affected patients are usually 50 to 70 years old (5), nevertheless, it has been found that as many as 19% could be younger than 40 years old and 7% younger than 30 (6).

There are multiple identified risk factors, such as phimosis, obesity, poor hygiene, history of multiple sexual partners, human papillomavirus (HPV) (genotypes 16 and 18), living in low-income areas, low level of schooling or no schooling, and smoking. Those are the most substantial factors associated with this illness (7, 8). Accordingly, the glans and foreskin chronic inflammations are considered a fundamental issue in physiopathology, leading to penile cancer (2). Other known factors are working in farming, being married, or in a stable relationship (4), and recently, a past of zoophilia has been considered an intriguing new factor (9). Circumcision in neonates is a protective factor; it reduces the risk by 70%. There is a low incidence of penile cancer in these patients, especially in Jewish and African tribes where circumcision is practiced as part of their religion (5, 8, 10, 11).

The diagnosis is clinical and histopathological, therefore, the nodule, ulcer, or mass biopsy should be a priority, preventing a treatment delay (7). Based on these findings, the patient will be classified with the TNM system for a more accurate treatment decision (12).

Every urologist must ensure making the treatment as effective and conservative as possible (13). There are multiple interventions to try to fulfill this objective, such as topic chemotherapy with imiquimod and 5-fluorouracil, laser with carbon dioxide or neodymium: yttrium-aluminum-garnet (Nd: YAG), glans resurfacing excision with circumcision laser, glansectomy with reconstruction, radiotherapy, partial amputation with reconstruction, radical penectomy with perineal urethrostomy, neoadjuvant or adjuvant therapy with surgery, among other interventions (14, 15). Also, there are different interventions for managing the inguinal region with the potential to limit morbidities, such as the dynamic sentinel node excision and the video endoscopic inguinal lymph node dissection (VEIL) (16, 17). T1 low-grade patients could be treated with a conservative approach; otherwise, higher T or high-grade stages will require more extensive procedures (1, 2, 18).

The low prevalence of penile cancer means that it might not meet the criteria for a screening campaign, for this reason, other strategies have been developed to prevent the disease, like gender-neutral HPV vaccination programs. Evidence exists that HPV vaccination of boys and men in a population in which girls and women already receive the vaccine would positively affect HPV-related disease (18), especially in the rural area because of the high incidence in this population (19).

Penile cancer is a low-incidence condition associated with high morbidity, impacting the patient’s functional and emotional aspects (20). So, involving people in health systems must prioritize these patients for early diagnosis and intervention. Timely attention prevents the disease progression and, in this way, decreases morbidity and mortality (21). It is well known that a diagnosis in the early stages reflects better preservation of sexual activity, prevents penectomy, and leads to better functional and cosmetic results because the conservative management depends on the disease stage (4).

An early penile cancer diagnosis and treatment are cornerstones for high survival and low morbidity. People living in poverty or rural areas with no appropriate knowledge of natural history and limited access to the health system will achieve higher TNM classification and progression. Hence, worse outcomes because they require more extensive and radical treatment (22). A low income is a significant predictor of health status, leading to more medical attention delays and participation in screening programs (23).

The previously exposed aspects significantly reflect in penile cancer higher morbimortality for people in rural areas (24, 25). After lesion detection, three months of medical attention delay directly relates to a more extensive compromise and a higher TNM classification (20). Besides, two years of impediments in attention have almost 100% of mortality (26). The rural population faces these delays and hence higher mortality.

From a different perspective, people living in places with higher than 20% of poverty have a 43% more risk of developing penile cancer than high-income places (27). Also, the risk of suffering invasive disease and morbimortality enhances (28).

On the other hand, the higher the TNM classification, the lower the survival at five years (33.3%, 40%, 100%, 80%, and 100% for stages IV, IIIb, IIIa, II, and I, respectively) (29). People living in a rural zone usually present with advanced disease, which means a lower survival and higher morbidity (8, 28). The previous might depend on poor knowledge, limited access to health services, and the associated genital stigma (24, 29).

Regarding the surgical treatment, delaying a lymph node dissection more than six months correlates with low survival (37.8%) at five years, compared with 77% survival when performing this procedure in less than three months (21).

Additionally, delays in lymphadenectomy correlate with a probability of 9.1% of local recurrence, which means survival in the next five years of only 1/3 of the patients (26). Because of this, a patient with lymph node enlargement or high-grade lesions should not delay its treatment since disease spread and metastasis predicts survival (30-32). People in rural areas frequently face the previous situation because of the limited access to the health system, which traduces in not timely attention and hence less survival.

The incidence of oncologic disease and mortality is higher in people living in rural areas when compared with the counterpart in the metropolitan area. There are more barriers in the attention associated directly with poverty, less access to the health system, remoteness from the hospital that can offer attention, and hence longer distances that need to be traveled for medical attention. Consequently, they have late diagnosis and treatments and lower outcomes when compared with the patients in the urban zones (33). HPV-associated cancer is more frequently found in rural areas where there is also limited vaccine access (34).

The HPV infection is a known factor for penile cancer. The worldwide incidence of HPV infection in males is heterogeneous, with an average of 50% incidence (35). Thirty to 50% of penile cancer cases are associated with HPV, especially genotypes 16 and 18 (36). In Brazil, a contemporary representative cohort of 1.132 men screened for HPV showed that over two-thirds are positive for human HPV DNA, 78% of high risk and over half with co-infections. The most frequently identified types were HPV-6, HPV-42, and HPV-16 (37).

People in rural areas have less knowledge about HPV infection, transmission, condom use, and access to the vaccine, therefore, the risk of penile and cervical cancer might increase. Forty percent of the rural people that knew about HPV infection did not know that it was related to cancer (38). There is an increased incidence of HPV infection in rural areas because of their sexual practices and vaccine access. They have less access to the health system, and less than 50% of them receive appropriate sexual education (39). For the above, the probability of consulting at later stages of the disease is enhanced, adding that the symptoms usually are initially ignored.

The uncircumcised people in the rural area have less access to the health system and poor hygiene, leading to chronic inflammation, so the risk of penile cancer augments (27, 34). Besides, the self-limitation belief of unspecific symptoms like eczema, erythema, induration, and ignorance about the malignancy potential, prevent seeking medical attention. Therefore, morbidity and mortality might increase. Once again reflecting the poor access to education leading to worse outcomes. So it is essential to avoid cognitive barriers in the search for better results (20).

In conclusion, penile cancer is a rare genitourinary malignancy with high morbimortality. There is an urge for preventive programs (40), timely diagnosis, and treatment with no delays since it brings functional loss (41), negatively impacting quality of life and survival. Belonging to a rural population with high poverty indices and low access to education and health care enhances the risk of worse outcomes because of the retard in the diagnosis and treatment, culminating with disease progression and spreading (42). Additionally, these individuals face other risk factors such as smoking, higher HPV infection prevalence, poor hygiene, unclear sexual patterns, which increase the risk of worst outcomes.

Since literature is still scarce, there is an urgent need to conduct more robust studies targeting environmental and behavioral aspects, especially in the rural areas, to advance the penile cancer understanding and allow specific actions targeting this vulnerable population, especially in the more unprotected zones.

## TAKE-HOME MESSAGES

Penile cancer is considered a rare genitourinary malignancy. The risk factors associated are phimosis, poor hygiene, human papillomavirus (HPV) infection, smoking, obesity, poverty, and living in the rural area. Circumcision in neonates is considered a protective factor.

The diagnosis is clinical and histopathological, so the physical exam and early biopsy of the ulcer or mass will define the treatment. In the early disease stages, the treatment is usually conservative, in contrast, more invasive interventions are required with more advanced diseases.

Early consultation delays of only three months are associated with more extensive lesions and higher TNM classification. It decreases the chance of conservative treatment and increases morbidity and mortality in these patients, with a five-year survival of only 33.3% for stages IV.

The population living in rural areas might go through different environmental and behavioral factors delaying diagnosis and treatment, such as accessing the health system, knowledge about the natural history, risky sexual intercourse, and higher prevalence of HPV infection. Accordingly, penile cancer needs an early diagnosis and treatment without delays.
